# Effect of microbial plant biostimulants on fruit and vegetable quality: current research lines and future perspectives

**DOI:** 10.3389/fpls.2023.1251544

**Published:** 2023-10-12

**Authors:** Francesca Melini, Valentina Melini, Francesca Luziatelli, Renée Abou Jaoudé, Anna Grazia Ficca, Maurizio Ruzzi

**Affiliations:** ^1^ CREA Research Centre for Food and Nutrition, Council for Agricultural Research and Economics, Rome, Italy; ^2^ Department for Innovation in Biological, Agrofood and Forest Systems (DIBAF), University of Tuscia, Viterbo, Italy

**Keywords:** plant biostimulants, plant growth-promoting rhizobacteria (PGPR), arbuscular mycorrhizal fungi, fruit quality, vegetable quality, sustainability, bioactive compounds

## Abstract

Fruit and vegetables hold a prominent place in dietary guidance worldwide and, following the increasing awareness of the importance of their consumption for health, their demand has been on the rise. Fruit and vegetable production needs to be reconsidered so that it can be productive and, meantime, sustainable, resilient, and can deliver healthy and nutritious diets. Microbial plant biostimulants (PBs) are a possible approach to pursuing global food security and agricultural sustainability, and their application emerged as a promising alternative or substitute to the use of agrochemicals (e.g., more efficient use of mineral and organic fertilizers or less demand and more efficient use of pesticides in integrated production systems) and as a new frontier of investigation. To the best of our knowledge, no comprehensive reviews are currently available on the effects that microbial plant biostimulants’ application can have specifically on each horticultural crop. This study thus aimed to provide a state-of-the-art overview of the effects that PBs can have on the morpho-anatomical, biochemical, physiological, and functional traits of the most studied crops. It emerged that most experiments occurred under greenhouse conditions; only a few field trials were carried out. Tomato, lettuce, and basil crops have been primarily treated with Arbuscular Mycorrhizal Fungi (AMF), while plant grow-promoting rhizobacteria (PGPR) metabolites were used for crops, such as strawberries and cucumbers. The literature review also pointed out that crop response to PBs is never univocal. Complex mechanisms related to the PB type, the strain, and the crop botanical family, occur.

## 1 Introduction

Fruit and vegetables hold a prominent place in dietary guidance worldwide, and a minimum consumption of 400 g (i.e., five servings) per person per day is recommended by the World Health Organization (WHO) and the Food and Agriculture Organization (FAO) (World Health Organization, 2020). Fruit and vegetables provide, in fact, the human body with an abundance of nutrients (i.e., vitamins, dietary fiber, micronutrients) and beneficial non-nutrient molecules such as bioactive compounds. Their intake has been shown to have an inverse correlation with the incidence of non-transmissible chronic diseases (NTCDs), such as cardiovascular diseases, cancer, obesity, and type 2 diabetes (Aune et al., 2017). Fruit and vegetables are the cornerstone of a healthy and varied diet (World Health Organization, 2020).

Following the increasing awareness of the important role played by fruit and vegetable consumption for the human health, their demand has increased. According to the FAOSTAT database, worldwide production of both fruit and vegetables rose by about half between 2000 and 2021: from about 572 to 909 million tons for fruits and from 687 to 1115 million tons for vegetables (FAO, 2023). Areas dedicated to fruit and vegetable cultivation have been thus extended (Hess and Sutcliffe, 2018). Nevertheless, the sustainability of their production methods must be investigated. In horticulture, the production systems are dominated by open field seasonal intensive crops with additive nutrient requirements to produce profitable marketable yields. In addition, also environments suffering from problems of water shortage are being used. Water supply, soil fertility, and pest and disease management are possible limiting factors for the sustainable production of fruit and vegetables.

Huge amounts of water are frequently required to maximize fruit and vegetable crops’ growth, yield, and quality. Climate, the irregular rainfall, wind and temperature patterns owed to climate change, or the competition with other human activities (e.g., domestic or industrial water use in urbanized areas) can impact the quantity and quality of available water (Hess and Sutcliffe, 2018). Drought may lead to water stress, limiting the plant’s ability to take up nutrients and resulting in a lower production of quality fruits and vegetables and, in the worst scenario, plant death (Hess and Sutcliffe, 2018). “Regulatory risks” of water scarcity also exist because of supply reallocation to sectors other than agriculture (i.e., higher priority sectors) in times of drought, as laid down by the European (EU) Water Framework Directive (European Commission, 2000). As to water quality, the sanitary status of the water used for fruit and vegetable production is of critical importance too. The use of water polluted by chemicals or heavy metals, urban waste, or sewage containing disease-causing microorganisms, as well as the salinization of freshwater resources, can have a detrimental effect (Thorslund and van Vliet, 2020).

Soil fertility and health can be further limiting factors to sustainable fruit and vegetable production. Soil fulfills, in fact, several functions. It can support plant growth by making nutrients available for root uptake; it stores and transforms compounds; it can filter, hold, and store fresh water; it prevents erosion. Soil fertility refers, therefore, to the ability of soil to maintain and sustain plant growth (Srivastava et al., 2021). Adequate salt and pH levels guarantee that the nutrients present in the soil are made available to crop plants. Evidence shows that moderately saline land is increasing yearly, limiting the possibilities for growing fruit and vegetables (Srivastava et al., 2021). A high salt concentration in the soil is detrimental for the plant as it reduces water uptake. Under salinity stress, transpiring leaves can be injured because salt enters the transpiration steam, and plant growth may be affected (Abdel-Farid et al., 2020). The decrease of water uptake and toxicity of sodium chloride may affect plant growth and thus reduce shoot length, leaf area and number (Abdel-Farid et al., 2020). Moreover, the root system is in direct contact with salt, which may harm the cell division of root tips and thus cause root length reduction.

Pest and disease management in fruit and vegetable crops is a significant issue too. Virus, bacterial, and fungi diseases and pests cause up to 40% yield losses in horticultural crops (Şener et al., 2020). For this, agricultural practices worldwide have long depended on the extensive and intensive use of chemical fertilizers and pesticides. The latter has, nevertheless, many undesirable aspects which should not be overlooked. They can remain in soil and environment for a long time and influence several biotic and abiotic factors. They adversely affect soil, microflora, other organisms, and the environment. In addition, residues remain in fruit and vegetables when, for instance, the time between the last spraying and harvest date is disregarded or when spraying over the recommended dose is carried out to achieve better results. Furthermore, pesticide residues (e.g., glyphosate, trifluralin, metazachlor, metamitron and sulcotrione, etc.) have accumulated in soils over the recent decades as “bound residues” with considerable ecotoxicological risk (Barriuso et al., 2008). This implies that their use can also be harmful to the human, animal, plant health and the environment.

Within this framework, fruit and vegetable production thus needs to be reconsidered to be productive and, at the same time, environmentally sustainable, resilient, and able to deliver healthy and nutritious diets. New technologies and approaches have been thus evaluated to achieve global food security and agriculture sustainability. The application of bio-based products, such as plant biostimulants (PBs), has thus emerged as a promising alternative to agrochemicals and a new frontier of investigation.

Plant biostimulants contribute to sustainable agriculture by exerting different beneficial effects for the plant growth and allow overcoming the detrimental effects of sub-optimal growing conditions on crops. In detail, PBs strengthen the plant root system architecture and biomass, boost nutrient absorption and utilization, increase photosynthetic activity, and improve plant tolerance to abiotic stresses, such as drought, extreme temperatures, salinity, and hypoxia (Ruzzi and Aroca, 2015; Carolina Feitosa de Vasconcelos and Helena Garófalo Chaves, 2019; Rouphael and Colla, 2020; Rakkammal et al., 2023). PBs also enhance crop quality, by fostering plant health and vigor, and increase harvestable yields. All the above thus allows for a reduction of fertilizer requirements, and this is of paramount importance in organic farming, where artificial fertilizers cannot be used.

To the best of our knowledge, no comprehensive reviews are currently available on the effects that microbial plant biostimulants’ application showed to have on specific horticultural crops. This review thus aims to provide a state-of-the-art overview of plant biostimulants’ application to fruit and horticultural crops, focusing on the benefits that microbial PBs can have on morpho-anatomical, biochemical, physiological, and functional traits of horticultural crops. To this aim, studies published in peer-reviewed journals and available on SCOPUS and Web of Science database were searched. Current research lines, challenges, and future perspectives of their application to horticulture are presented and discussed. Where available, the impact of biostimulants on plants via genomic, proteomic, and transcriptomic changes, is also analyzed.

## 2 Plant biostimulants

Plant biostimulants are defined in Regulation (EU) No. 2019/1009 as products “stimulating plant nutrition processes with the sole aim of improving: i) nutrient use efficiency; ii) tolerance to abiotic stress; iii) quality traits; and iv) availability of confined nutrients in soil or rhizosphere” (European Union, 2019). The Regulation also makes a distinction between microbial and non-microbial PBs. A microbial PB is a microorganism or a consortium of microorganisms, and this term applies only to Arbuscular Mycorrhizal Fungi (AMF) and Plant Growth-Promoting Rhizobacteria (PGPR) belonging to the *Azotobacter*, *Azospirillum*, and *Rhizobium* taxonomic groups, according to the current, but extendable, positive list in the Annex of EU Regulation No. 2019/1009 (European Union, 2019). A non-microbial plant biostimulant is a plant biostimulant other than a microbial PB. It encompasses humic substances (e.g., fulvic and humic acids), animal- or vegetal-based protein hydrolysates, seaweed extracts, and botanicals (Ruzzi and Aroca, 2015; Yakhin et al., 2017; European Union, 2019).

Among microbial PBs, AMF are soil fungi forming a mutualistic symbiosis with the roots of plants (Rouphael et al., 2015). In the presence of a host plant, AMF spores germinate, and a hyphal structure link between the plant and the fungus is formed. AMF can benefit the plant in case of abiotic stresses, such as drought, salinity, nutrient deficiency, adverse soil pH, and heavy metals. In drought, AMF creates hyphae on plant roots, implying that AMF increase the extension of the host’s root system and contribute to plant growth by linking the plant and the immobile nutrients in the soil. The plant’s nutrient uptake and water absorption are enhanced (Ebbisa, 2022). AMF’s ability to increase plant root surface area also affects the ability of AMF to secure greater plant productivity and yield stability by enhancing nutrient uptake and promoting stress tolerance. In addition, the symbiosis between the plant and AMF has been shown to influence secondary metabolism by enhancing the synthesis of phytochemicals (Rouphael et al., 2015). This aspect is pivotal in obtaining horticultural products with improved nutraceutical value.

Plant Growth-Promoting Rhizobacteria (PGPR) have also become a significant body of research. They include strains in the genera *Agrobacterium*, *Arthrobacter*, *Azospirillum*, *Azotobacter*, *Bacillus*, *Burkholderia*, *Caulobacter*, *Chromobacterium*, *Enterobacter, Erwinia*, *Flavobacterium*, *Micrococcus*, *Pantoea*, *Pseudomonas* and *Serratia* (Verma et al., 2019; Luziatelli et al., 2020a; Luziatelli et al., 2020c; Luziatelli et al., 2021). This heterogeneous group of endophytic and epiphytic bacteria, which dwell in association with plant tissues and surfaces, can positively affect plant health and growth (Glick, 2010; de Souza et al., 2015). As any PBs, they can increase crop tolerance against abiotic stresses (Wu and Zou, 2009; Ruzzi and Aroca, 2015; Colla et al., 2017; Rouphael and Colla, 2018; Woo and Pepe, 2018) and improve nutrient use efficiency, photosynthetic activity, as well as plant health, growth, productivity and yield at different stages (Bulgari et al., 2015; Toscano et al., 2018). PGPR exert the aforesaid beneficial effects on plants through direct and indirect mechanisms. They can specifically interact with plants directly by making essential nutrients (e.g., nitrogen, phosphorus, iron) more available, by producing and regulating some compounds involved in plant growth (e.g., phytohormones), and by affecting the hormonal status stress (e.g., ethylene levels by ACC-deaminase). PGPR can also promote plant growth by inducing the expression of auxin-responsive genes in host-plant roots without producing these hormones and only by the auxin signaling pathway (Ruzzi and Aroca, 2015). The production of auxins elicits transcriptional changes in hormone and cell wall-related genes, induces longer roots, increases root biomass and decreases stomata size and density (Backer et al., 2018). In addition, PGPR can indirectly affect plant growth, which according to the current EU regulations is a trait not to be claimed for commercial plant biostimulant products, even though it is well known from the scientific literature. A legal regulation that is more according to the nature of the regulated entities therefore would be necessary to provide more adequate recommendations for their use in crop production. PGPR can protect plants against diseases, by competing with pathogens for minimal nutrients, exerting a biocontrol action of pathogens through the production of aseptic-activity compounds, synthesizing fungal cell wall lysing enzymes, and inducing systemic responses in host plants (Oleńska et al., 2020). PGPR potential to ease plant growth is therefore of paramount importance, also in case of abiotic stresses, because bacteria can enhance plant fitness, mitigate stress tolerance, or even assist in the remediation of pollutants.

## 3 Current research lines on the effect of microbial plant biostimulants on fruit and vegetable quality

The principal crop types, as treated by PBs in general, include row crops (cereals, oilseeds, pulses, and fiber crops), fruit and vegetables, turf ornamentals, and “others” (Critchley et al., 2021). However, the analysis performed within this study showed that the effect of microbial PBs has been so far studied mainly on horticultural crops, such as tomato (*Solanum lycopersicum* L.), cucumber (*Cucumis sativus* L.), strawberry (*Fragaria* × *ananassa* Duch.), rocket (*Eruca vesicaria* Mill.), lettuce (*Lactuca sativa* L.), and basil (*Ocimum basilicum* L.). Experimental microbial/fungal PBs have been applied in pot, greenhouse, and field experiments through seed/root inoculation, soil treatment, foliar application, or microbial amendments.

Current research lines followed up a literature search performed in Scopus, Web of Science and PubMed databases. Studies published from 2018 to 2022 were considered for inclusion in the analysis. The following list of search terms was used: plant biostimulants, plant growth-promoting (rhizo)bacteria, Arbuscular Mycorrhizal Fungi, field experiments, greenhouse, horticultural crops, food quality, sustainability, bioactive compounds, omic sciences, transcriptomic, genomics, proteomics. This list derives from a preliminary set of scoping searches conducted to test out search terms and find possible additional terms to design the search strategy.

### 3.1 Tomato

Tomato (*Solanum lycopersicum* L.) is one of the most widely grown vegetables, with a global production of almost two hundred million tons covering a harvested area of about five million hectares in 2021. Tomato is consumed fresh and processed as pulp or sauce. Its fruits are a good source of dietary minerals and especially contain trace elements, such as iron, zinc, copper, calcium, potassium, and magnesium; they are also rich in vitamins C and E, folic acid and other organic acids (e.g., malic, ascorbic, and citric acid), and in essential amino acids (e.g., leucine, threonine, valine, histidine, lysine, and arginine) (Chaudhary et al., 2018). Tomato is also a source of bioactive compounds: carotenoids (i.e., lycopene and β-carotenoids), phytosterols, and phenolic compounds (e.g., quercetin, kaempferol, naringenin, caffeic acid) (Chaudhary et al., 2018). Bioactive compounds contribute to tomato antioxidant, anti-inflammatory, anti-mutagenic, anti-proliferative, and anti-atherogenic activity (Chaudhary et al., 2018), and make tomato an essential food of a healthy diet, such as the Mediterranean one (Naureen et al., 2022).

Tomato cropping is adapted to a wide range of growing conditions; however, worldwide productivity faces the challenge of biotic and abiotic stress. Sub-optimal temperatures after transplanting, poor soil fertility, and excessive temperature during flowering and fruiting can strongly reduce crop productivity (Cardarelli et al., 2020). Hence, an extensive use of fertilizers is required to overcome the aforesaid issues and obtain optimal plant growth and high-quality yields. Drought and salinity and various pathogens and pests are also limiting factors (Bai et al., 2018).

In identifying new approaches to implement sustainable agriculture, efforts have been made to apply PBs to tomato crops to boost yield, fruit quality, and production sustainability under environmental conditions which may limit crop growth. In addition, given this crop’s commercial and nutritional value, efforts have been made to regulate the plant physiology so that the biochemical composition of the fruit, including the content of bioactive components, such as vitamin C, lycopene, among others, is also improved.

Several studies have been published over the last five years where tomato seedlings and plants were treated by seed/root inoculation, direct soil treatment, foliar application, and with PGPR strains, AMF, or a combination thereof. The effect of treatments was evaluated on different target parameters, ranging from plant morphology and crop productivity to the functional and sensory quality of the fruits (**Table 1**).

**Table 1:** Conditions and effect of microbial plant biostimulants treatment on tomato crops.

Crop	Growing conditions and location	PGPR	AMF	Treatment effect on targeted parameter(s)		Reference
Tomato (*S. lycopersicum* L., var. CXD 219 F1)	Field experiment (Italy)	*Pseudomonas* sp. 19Fv1T *Pseudomonas fluorescens* C7	AMF mix	Plant collar diameter	=	(Bona et al., 2018)
				% fruits/flowers	=	
				Fruit length	↑	
				Fruit diameter	↑	
				Fruit weight	↑	
				Lycopene	↑	
				β-carotene	↑ (2 out of 3)	
				Lutein	↑ (2 out of 3)	
Tomato (*S. lycopersicum* L. cv. PS1313)	Open field experiment (Italy)	–	Mycorrhizal fungi and *Trichoderma*	SPAD index	=	(Cardarelli et al., 2020)
				Marketable yield	↑	
				Immature yield	=	
				Unmarketable yield	=	
				Fruit fresh weight	↑	
				TSS	=	
				Juice pH	=	
				N	↑	
				P	=	
				K	=	
				Vitamin C	↑	
				Lycopene	↑	
Cherry tomato (*S. lycopersicum* L. cv. Pomodorino del Piennolo del Vesuvio)	Field experiment (Italy)	–	*Rhizoglomus irregulare* *Funneliformis mosseae*	Yield	↑	(Carillo et al., 2020)
				Mineral profile	↑ (P, Ca, Mg, Na, Cu, and Zn)	
				TSS	↑	
				Fruit dry matter	↑	
				TAA	↑	
				Lycopene	↑	
				Total Ascorbic Acid	↑	
Tomato (*S. lycopersicum* L. cv. Kwangbok)	Greenhouse experiment (Korea)	*Bacillus subtilis* CBR05	–	TAA	↑	(Chandrasekaran et al., 2019)
				TPC	↑	
				TFC	↑	
				All-E-β-Carotene	=	
				All-E-Lycopene	↑	
Tomato (*S. lycopersicum* L. cv. Rio Fuego)	Greenhouse experiment (Mexico)	–	*Rhizophagus intraradices*	Aerial part	↑	(González-González et al., 2020)
				Total carbohydrate	↑	
				Total protein	↑	
				TPC	↑	
Tomato (*S. lycopersicum* L. cv. Rio Grande)	Field experiment (Greece)	*Bacillus amyloliquefaciens* B002 *Bacillus licheniformis* B017 *Bacillus mojavensis* B010 *Bacillus pumilus* W27-4 *Bacillus subtilis* Z3 *Bacillus pseudomycoides* S3 *Bacillus velezensis* B006 *Azotobacter chroococcum* A004 *Bacillus megaterium* B004	–	Plant dry weight	↓↑	(Katsenios et al., 2021)
				Photosynthetic Rate	↓↑	
				Transpiration Rate	↓↑	
				Mean fruit weight	↑ (5 out of 10)	
				Yield/plant	↑ (9 out of 10)	
				TSS	↑ (10 out of 10)	
				PME Activity	↑ (8 out of 10)	
				PG activity	↑ (5 out of 10)	
				TCC	↑ (6 out of 10)	
				TPC	=	
				Lycopene	↑ (5 out of 10)	
				TAA	↑ (4 out of 10)	
Tomato (*S.lycopersicum* L. cv. Marmande)	Pot experiment (Italy)	*Pantoea agglomerans* C1	–	Root system	↑	(Luziatelli et al., 2020b)
Tomato (*S. lycopersicum* L. cv. San Marzano)	Greenhouse experiment (Italy)	–	–	Marketable yield	↑	(Rouphael et al., 2021)
				Shape index	=	
				Juice pH	↑	
				Starch	↑ (1 out of 4)	
				Soluble sugars	↑ (1 out of 4)	
				Anthocyanins	=	
				Lycopene	↑	
				Polyphenols	↑	
				Soluble proteins	=	
				Free Amino Acids	=	
Tomato (*S. lycopersicum* L. cv. San Marzano)	Greenhouse experiment (Italy)	*Micrococcus* sp. F3 *P. agglomerans* C1 *Pseudomonas* sp. F1G	*Rhizophagus irregularis*	Root growth	↑ (PGPR; low P) ↑ (AMF; low+high P)	(Saia et al., 2020)
				Root biomass	↑ (PGPB; AMF; high P)	
				Total Root length	↑ (PGPB; AMF; high P)	
				Total Root Area	↑ (PGPB; low P) ↑ (PGPB; AMF; high P)	
				Leaf biomass	↑ (PGPB; AMF; high P)	
				No. of leaves	↑ (PGPB; AMF; high P)	
				Leaf area	↑ (PGPB; AMF; high P)	
				No. flowers	↑ (AMF; high P)	

AMF, Arbuscular Mycorrhizal Fungi; Ca, calcium; Cu, copper; Mg, magnesium; N, nitrogen; Na, sodium; P, phosphorous; PGPR, Plant Growth-Promoting Rhizobacteria; SPAD, Soil Plant Analysis Development; TAA, Total Antioxidant Activity; TCC, Total Carotenoid Content; TPC, Total Phenolic Content; TSS, Total Soluble Solids; Zn, zinc; ↑, increase; ↓, decrease; ↑↓, variable; =, no significant differences.

#### 3.1.1 Effect of microbial PBs on tomato plant and fruit morphology and crop productivity

Tomato quality is generally defined by several physical-morphological traits, such as plant rooting system, number of flowers and fruits, fruit shape, length, diameter and weight, and total soluble solids. They are affected by many biochemical mechanisms, at the plant and fruit level, which depend on the interaction between cultural practices and genetic and environmental factors. The analysis of studies investigating the effect of microbial treatment on plant morphology and crop productivity has shown that these traits have been studied in different cultivars (e.g., *Solanum lycopersicum* L. cv. San Marzano, cv. Rio Grande, etc.) under different growing conditions.

The combined effect of mycorrhizal fungi, *Trichoderma*, and non-microbial PBs (i.e., vegetal extracts) on tomato crop productivity was investigated (Cardarelli et al., 2020). Treatments did not significantly affect the Soil Plant Analysis Development (SPAD) index, which is a non-destructive measurement of leaf chlorophyll content and an indicator of the photoinhibition of the Photosystem II (PSII); however, the general good health status of the crop and good photosynthetic activity of the tomato plants were observed after the PB treatment, compared to non-treated plants. Regarding yield, the total marketable yield was significantly higher in PB-treated plants. At the same time, no significant differences were observed for immature yield and unmarketable yield factors (e.g., blossom end rot fruits and rotten fruits; **Table 1**). The increase in marketable yield was related to a greater mean fruit weight than a change in the number of tomato fruits. Tomato growth was stimulated by both AMF and the ability of *Trichoderma* to release auxin-like compounds in the rhizosphere. The latter can solubilize mineral nutrients, such as phosphorus and micronutrients (e.g., Fe), and control plant pathogens through antagonistic activity and induction of systemic resistance in plants (Cardarelli et al., 2020). The effect of treatments on fruit quality was not univocal; an 18% increase was observed for fresh tomato weight, while total soluble solids and tomato juice pH were not significantly affected.

The same parameters as Cardarelli et al. (2020) were investigated by Rouphael et al. (2021) in a greenhouse experiment, where four different tomato landraces of San Marzano cultivar were treated with a plant extract (by foliar application). The application of the plant extract had a little positive effect on yield, whose increase varied among landraces. The growing conditions did not affect the shape index, calculated as a ratio of the maximum height length to maximum width relative to the longitudinal section (**Table 1**).

A microbial-based biostimulant containing *Rhizophagus intraradices* was applied to tomato plants cv. ‘Rio Fuego’ i) alone (AMF), ii) in combination with irrigation with a nutritive solution (AMF+NS), and iii) with the nutritive solution and a non-microbial-based biostimulant from *Padina gymnospora* extract (AMF+NS+SE) (González-González et al., 2020). As regards root mycorrhizal colonization, an established mycorrhizal symbiosis was observed 96 days after treatment (DAT) in the AMF-treated and AMF+NS+SE plants, with a higher abundance in the second group of plants. The physiological response of tomato plants to the various applications showed that the AMF treatment favored the growth of the aerial plant. Moreover, the high polyphenol content, non-photochemical quenching, and maximum photochemical quantum yield efficiency of PSII in a dark-adapted state (F_V_/F_M_ values) implied that AMF conferred the plant high resistance to environmental stress, as well as an increase in antioxidant and photoprotective mechanisms. The AMF biostimulant activity is related to the root biomass regulation, which may enhance nutrient uptake and translocation, increasing total carbohydrate, protein, and phenolic content (**Table 1**), as well as in plant-growth promotion, biomass production, stress tolerance, and disease resistance (González-González et al., 2020). The hyphal networks produced by AMF improve soil quality by increasing soil particle aggregation and reducing soil erosion. AMF also limits the amount of nutrient leaching from the soil and promotes nutrient retention (Tavarini et al., 2018). The combination of AMF, nutritive solution, and seaweed extracts allowed for an additive effect and increased foliar and root growth and protein and carbohydrate content.

A commercial microgranular inoculum containing *Rhizoglomus irregulare* and *Funneliformis mosseae* spores was placed in the planting hole of two landraces of Pomodorino del Piennolo del Vesuvio cherry tomatoes, i.e., the yellow-pigmented type “Giagiù” and red-pigmented type “Lucariello” (Carillo et al., 2020). Mycorrhization showed a beneficial effect on fruits. The number thereof per plant increased significantly, but no positive effect was observed on the mean fruit mass (**Table 1**). The capacity to accumulate starch was also augmented in both landraces. This is due to an increase of the photosynthetic efficiency and free amino acids. The positive effect of AMF on amino acids profiling depends on the beneficial effect of symbiosis on nutrient availability. AMF can, in fact, directly solubilize plant nutrients in the soil rhizosphere or produce siderophores; thus nutrients are directly available to the plant (Carillo et al., 2020).

Flower and fruit production was assessed in tomato (*S. lycopersicum* L., var. CXD 219 F1) plants grown in a field experiment under six conditions (Bona et al., 2018). It emerged that the treatments with the mycorrhizal inoculum (containing *Rhizophagus intraradices*, *Rhizophagus aggregatus*, *Septoglomus viscosum*, *Claroideoglomus etunicatum*, and *Claroideoglomus claroideum*), *Pseudomonas* sp. 19Fv1T, and *Pseudomonas fluorescens* C7 did not significantly affect the collar diameter and the percentage of fruits and flowers. The effect of the treatments on traits, such as the number of flowers and fruits, the total weight of mature fruits, and the percentage of non-marketable fruits, was not univocal (**Table 1**). Inoculation with the selected soil microorganisms significantly increased fruit size (length and diameter) and weight. This outcome is of paramount importance, since producing larger and heavier fruits is of high economic interest.

AMF has also been used with PGPR strains to investigate a possible synergistic action of the two categories of microbial PBs (Saia et al., 2020). AMF and three different strains of PGPR (i.e., *Micrococcus* sp. F3, *Pantoea* sp. C1, *Pseudomonas* sp. F1G) were applied by Saia et al. (2020) in a greenhouse experiment where tomato plants were grown under two different fertilizing conditions: low and high phosphorous. In this work, the authors evaluated the effect of the different treatments on roots biomass and traits (e.g., total length, specific length, mean diameter, total area, specific area), and leaf biomass and area, number of leaves and flowers. Whatever PGPR strains were applied, an increase in the root growth was observed under conditions of low phosphorus availability; on the other hand, AMF boosted root growth regardless of the fertilizer type. In detail, the treatment with *Pantoea* sp. C1 was the most promising, with a 121% increase in the total root length under low phosphorus conditions; *Micrococcus* sp. F3 determined an increase in the total root length as well, which was higher under low phosphorus conditions than under high phosphorus conditions; the AMF treatment allowed for an increase of total root length. The PGPR treatment also improved leaf area, aboveground and root biomass, and nitrogen concentration but only under low-phosphorous conditions, that is, when Gafsa rock phosphate was used. This is related to PGPR’s ability to produce root branching hormones, especially for the *P. agglomerans* C1 (Luziatelli et al., 2019). AMF improved the SPAD index, the total root area, and nitrogen concentration.

*P. agglomerans* C1 cells and metabolites were applied to tomato seedlings in pot experiments (Luziatelli et al., 2020b). The effect of the treatment on root characteristics was compared to i) sterilized distilled water (control), ii) sterilized distilled water supplemented with LB medium, iii) spent medium containing cells, iv) cell-free spent medium, and v) indole-3-butyric acid solution. When tomato shoots were treated with the spent medium containing both cells and secreted metabolites, a significant increase in the root surface area was observed, compared to the control, that is, the shoots treated with distilled water (**Table 1**). When the cell-free spent medium was applied, a more remarkable root growth was observed. The application of the extracellular metabolites produced by strain C1 determined an increase in the number and length of main roots of tomato cuttings; this indicates that strain C1 produces metabolites which can boost plant growth.

The effect of nine PGPR (i.e., *Bacillus amyloliquefaciens* B002, *Bacillus licheniformis* B017, *Bacillus mojavensis* B010, *Bacillus pumilus* W27-4, *Bacillus subtilis* Z3, *Bacillus pseudomycoides* S3, *Bacillus velezensis* B006, *Azotobacter chroococcum* A004, *Priestia megaterium* B004) and one mix thereof, on plant growth and physiology, yield, and quality characteristics of tomato fruits, cv. ‘Rio Grande’, was evaluated in a field experiment in Greece (Katsenios et al., 2021). The study outcomes showed that the soil application of the PGPR improved the industrial tomato’s plant growth and physiology, yield, and quality characteristics. The effect of the treatments on dry weight, mean fruit weight and yield per plant, as well as on photosynthetic and transpiration rate was monitored at 66, 80, and 94 DAT. At the final measurement, *B. licheniformis* and *B. subtilis* treatments determined an increase in dry weight per plant of about 39.38% and 32.23%, respectively (**Table 1**). The application of *P. megaterium* boosted the photosynthetic rate from 5.54% at 66 DAT to 25.73% at 94 DAT; however, the highest values at the final measurement were observed in the treatment with *B. velezensis* and *A. chroococcum*. After 80 DAT, the treatment with the PGPR strains notably enhanced the transpiration rate, except for *B. amyloliquefaciens*, *A. chroococcum*, and *P. megaterium*.

#### 3.1.2 Effect of microbial PBs on tomato functional and sensory quality

Food quality is a multi-faceted concept defined by several aspects related to commodity quality, outlined by external food attractiveness (e.g., fruit form, size, color), firmness, shelf-life, and organoleptic and functional quality. In tomato fruits, organoleptic quality is defined by physical traits, such as texture or firmness, and by biochemical traits (e.g., sugar and organic acid content and volatile compounds) determining the overall flavor; functional quality is defined by vitamins, phytonutrients (e.g., lycopene, β-carotene, polyphenols and ascorbate) and minerals (e.g., potassium, calcium, magnesium and phosphorus). As shown in **Table 1**, lycopene is the most studied parameter to evaluate the effect of PBs treatment on tomato functional quality.

Cardarelli et al. (2020) observed that inoculation with AMF and *Trichoderma koningii* combined with vegetal extracts determined a 14.1% increase in lycopene content and a 6.1% increase in vitamin C (**Table 1**). It was speculated that AMF may have promoted the activity of key enzymes involved in antioxidant homeostasis in cells. In addition, AMF can modulate host plant primary and secondary metabolism and stimulate the synthesis and accumulation of phytochemicals. Mineral concentration in tomato fruits was also investigated, and it emerged that concentration is affected positively only in the case of nitrogen; for phosphorus and potassium, no significant (p>0.05) differences were observed.

In the study by Rouphael et al. (2021), where four landraces of San Marzano were treated with a plant extract in a greenhouse experiment, lycopene, simple sugars, some organic acids, and macro-elements were investigated in tomato fruits. Lycopene concentration significantly increased following the PB treatment (**Table 1**). This result is paramount because lycopene content in mature fruits is important from a functional point of view and a critical aspect of the processing step. The increase in lycopene content determines, in fact, an increase in the fruits’ red color intensity. Polyphenols, especially anthocyanins, did not vary between the control and the treated samples. As regards sugars, which are a crucial parameter as they are key contributors to tomato flavor, it was observed that the application of the plant extract positively affected glucose and fructose but not sucrose content. The biostimulation by the plant extract did not affect total protein content and free amino acids, including the essential. Citric acid increased in all landraces treated with the plant extract, but one. Either genotype or biostimulants influenced the mineral profile of tomato fruits. Interestingly, the effect of biostimulation was mineral-specific; it increased, in fact, the fruit concentration of Mg and K cations, while it decreased the concentration of the NO_3_ anion. These data are important regarding the fruit’s nutritional value because the abovementioned elements are essential minerals (Rouphael et al., 2021).

Carillo et al. (2020) also studied the mineral profile in the two Pomodorino del Piennolo del Vesuvio cherry tomato landraces. It was observed that AMF inoculation determined a significant increase in the content of magnesium, phosphorous, calcium, sodium, cuprum, and zinc (**Table 1**). The highest calcium and sodium concentrations in tomato fruits, for which a significant effect was determined by the interaction of landrace and microbial-based biostimulants, were observed in the “Giagiù” landrace. The higher calcium concentration observed in Giagiù after treating AMF is a particularly relevant trait because calcium positively affects morpho-physiological and metabolic parameters. It contributes, in fact, to increasing fruit number and yield, as well as to maintaining the firmness and turgor of fruit tissues and to extending fruit shelf-life. The increase in zinc concentration is important because it is beneficial not only for the plant and fruits but also for humans. The ion is, in fact, essential for protein and starch synthesis; it is involved in superoxide radicals scavenging and contributes to the integrity of membranes. It regulates the expression of genes participating to cell expansion and is crucial to the correct development of tomato fruits. Zinc is finally essential for human nutrition, because it plays a key role in the growth, differentiation, and metabolism of cells. Hence, an adequate intake is crucial during human growth, e.g., prenatal and infancy stages. The effect of AMF treatment on lycopene and total ascorbic acid of the studied landraces was also investigated. The “Lucariello” landrace showed an 85% increase in lycopene compared to commercial tomatoes. Inoculation with endophytic fungi also enhanced total ascorbic acid, for which a higher content was observed (**Table 1**). The increase in lycopene and total ascorbic acid implies that fruit tissues are more protected from oxidative stress, and the postharvest conservation of “Lucariello” tomatoes can be extended. In addition, ascorbic acid plays a key role in maintaining fruit color and freshness, and in preventing spoilage, principally because of its antioxidant activity and low pH that limits food spoilage. AMF inoculation averaged over landrace positively affected the total soluble solids, fruit dry matter percentage, total antioxidant activity, and lycopene in fruit tissue (**Table 1**).

The effect of treatment with *B. subtilis* CBR05 on phytochemicals was investigated by Chandrasekaran et al. (2019). The greenhouse experiment showed that the antioxidant activity, measured by the DPPH and ABTS scavenging capacity, was positively affected by applying the PGPR strain. An increase in total phenolic content and total flavonoid content in treated plants was also observed (**Table 1**). A significant (p<0.05) higher amount of lycopene (All-E-lycopene) was also found, while no significant differences compared to the control were observed for All-E-β-Carotene (Chandrasekaran et al., 2019).

Bona et al. (2018) monitored the effect of treatment with AMF, *Pseudomonas* sp. 19Fv1T, and *P. fluorescens* C7 on glucose and fructose concentrations, and it emerged that they were highest when a combination of the three PBs was applied. A 13% and 19% increase in glucose and fructose, respectively, were found (**Table 1**). This finding is very important because the sweetness is a very appreciated characteristic in tomatoes for industrial use, and fructose is the most important sugar for the sweetness perception because it is rated at 1.7 times the sweetness of sucrose.

Tomatoes treated with the AMF *R. irregularis* and *F. mosseae* under optimal and low nitrogen (N) input conditions, in combination with *Trichoderma atroviride* application or alone, showed an enhanced functional quality of fruits (Ganugi et al., 2023). AMF coupling with *Trichoderma* fungal inoculations resulted in a synergistic effect on tomato fruits under sub-optimal fertility conditions. The concentration of β-carotene, Z-carotene, 13-Z-lycopene, and all-trans-lycopene increased; total phenolic content, total antioxidant activity, radical scavenging activity, reducing power and enzyme inhibitory activity were also boosted when AMF was applied with *T. atroviride* in combination with low N input and under sub-optimal fertility conditions (**Table 1**). These results point out that AMF inoculation helps plant growth under low nitrogen conditions by modulating the response thereof at physiological, metabolic, and molecular levels, accumulating secondary metabolites in the host plant.

### 3.2 Strawberry

Strawberry (*Fragaria* × *ananassa* Duch.) is the most consumed berry fruit crop worldwide, and its quality is defined by a unique flavor, its nutrients, and the antioxidant potential. Appearance, in terms of shape, color, size, and gloss; texture, in terms of firmness, crispness, and toughness; flavor, in terms of sweetness, sourness, aroma, and off-flavors; nutritional profile, in terms of vitamins and minerals; antioxidants, in terms of anthocyanins, flavonols and flavanols, phenolic acids, carotenoids, and vitamins, are quality indices important to evaluate strawberry quality (Giampieri et al., 2012; Cheng et al., 2016; Miller et al., 2019).

Strawberry (*Fragaria* × *ananassa* Duch.) cropping nevertheless requires extensive use of fertilizers at various stages of the plant life cycle. This use is detrimental in a framework of economy, environment, and human health safeguards. Recent studies have thus investigated the potential of PGPR application as an alternative to chemical fertilizers and their effect on strawberry fruit’s nutritional and organoleptic quality. The application of PGPR to strawberry cropping can have valuable environmental benefits: it may help to reduce the use of chemical fertilizers, it can suppress phytopathogens, and it may help to sustain soil productivity.

#### 3.2.1 Effect of microbial PBs on strawberry plant and fruit morphology and crop productivity

The effect of microbial PBs on strawberry plant and fruit morphological traits has been deeply investigated (**Table 2**). Fruit length, width, and sphericity have been widely analyzed; plant morphology was measured in a few cases.

**Table 2:** Conditions and effect of microbial plant biostimulants treatment on strawberry crops.

Crop	Experiment Type and Location	PGPR	AMF	Quality parameter	Treatment Effect	Reference
Strawberry (*Fragaria* × *ananassa* Duch. cv. Honeoye)	Pot experiment (Poland)	Mixed consortia of *Peanibacillus polymyxa* sp., *B. subtilis*, *Bacillus* sp., *Streptomyces* sp., *Lysobacter* sp., and *Pseudomonas* sp.	–	Firmness	↑ (1 out of 5)	(Drobek et al., 2021)
				SSC	↑ (1 out of 5)	
				TAC	↑ (4 out of 5)	
				TPC	↑ (1 out of 5)	
Strawberry (*Fragraria* x *ananassa* Duch. var. Camarosa)	Greenhouse experiment (Spain)	*Phyllobacterium endophyticum* (type-strain)	–	Stolons number and length	↑	(Flores-Félix et al., 2015)
				Flowers/Fruit number	↑	
				Fruit weight	=	
				Vitamin C	↑	
Strawberry (*Fragaria* × *ananassa* Duch. var. Camarosa)	Greenhouse experiment (Portugal)	*Pedobacter* sp. CC1, *Bacillus safensis* B106, *B. subtilis* B167A	–	Flowering	↑	(Morais et al., 2019)
				Harvest time	↑	
				Fruiting season	↑	
				Leaves number/plant	=	
				Plant height	=	
				Fruit color	=	
				Fruit length	↑ (*B.subtilis, Pedobacter* sp.)	
				Fruit width	=	
				Fruit sphericity	=	
				Chlorophyll content	=	
				TCC	↓	
				TPC	↑ (all treatments)	
				TFC	↑ (*Pedobacter* sp.)	
				TAC	=	
				AA	↑ (*B.subtilis, Pedobacter* sp.)	
Strawberry (*Fragaria* × *ananassa* Duch. cv. Hood)	Field experiment (Oregon, USA)	*B. subtilis* *Bacillus amyloliquefaciens* *Pseudomonas monteilii*	–	TA	↓ (T1) =(T2)	(Nam et al., 2023)
				TSS	= (T1) ↑(T2)	
				Color (L)	↓ (T1, T2)	
				Color (chroma)	↓ (T1, T2)	
				VOCs	↑	
Strawberry (*Fragaria* × *ananassa* Duch. cv. Festival)	Field experiment (Bangladesh)	*B. amyloliquefaciens* BChi1 *Paraburkholderia fungorum* BRRh-411	–	Root length	↑	(Rahman et al., 2018)
				Plant height	=	
				Leaf length	↑	
				Leaf width	↑	
				Leaves number/plant	↑	
				Canopy diameter	↑	
				Fruit length	↑	
				Fruit diameter	↑	
				Fruit weight	↑	
				TAC	↑	
				TCC	↑	
				TFC	↑	
				TPC	↑	
				AA	↑	
Strawberry (*Fragaria* × *ananassa* Duch. var. Eliana F1)	Greenhouse experiment (Italy)	*Pseudomonas* sp. (19Fv1t, 5Vm1K and Pf4	*F. mosseae* *S. viscosum* *R. irregularis*	Flowers/plant	↑ (Pf4)	(Todeschini et al., 2018)
				Fruits/plant	↑ (Pf4)	
				Total fruit fresh weight/plant	↑ (Pf4)	
				Average weight of fruit/plant	↑	
				Fruit large diameter	↑	
				Fruit small diameter	↑	
				pH	↑↓	
				Titratable acidity	↑	
				Malic acid	↑	
				Quinic, citric, fumaric and ascorbic acid	=	
				Sucrose	↑↓	
				Glucose	=	
				Fructose	↑	
				Total sugar concentration	=	
				Total anthocyanidin	↑ (*S. viscosum*+5Vm1K)	
				Pelargonidin 3-glucoside concentration	↑ (*S. viscosum*+5Vm1K)	

AA, antioxidant activity; GAC, Galacturonic Acid Content; SSC, Soluble Sugar Content; TA, total acidity; TAC, total anthocyanins content; TCC, total carotenoid content; TFC, total flavonoid content; TPC, total phenolic content; TSS, total soluble solids; VOCs, volatile compounds; ↑, increase; ↓, decrease; ↑↓, variable; =, no significant differences.

The effect of strawberry plant treatment with PGPR strains *B. amyloliquefaciens* BChi1 and *Paraburkholderia fungorum* BRRh-411 was investigated in field conditions (Rahman et al., 2018). *B. amyloliquefaciens* is a Gram-positive spore-forming bacterium in soil, which can colonize plant rhizosphere and grow under stressed conditions. It has been studied as an eco-friendly and non-toxic agent able to stimulate plant growth without having detrimental side effects (Luo et al., 2022). *P. fungorum* is a Gram-negative environmental species commonly used as a beneficial microorganism in agriculture and as an agent for biocontrol and bioremediation. Nevertheless, its application in agriculture is controversial because there is no clear evidence about its non-harmfulness to human health. The application of the two strains in field experiments showed a beneficial effect on plant, leaf and fruit morphology and fruit antioxidant content (**Table 2**) (Rahman et al., 2018). Results showed that apart from plant height, for which no significant (p > 0.05) difference was observed between the control and the treatments (**Table 2**), the two PGPR determined a significant increase in all the investigated parameters: rooting apparatus, number of leaves per plant, plant length and width, and canopy diameter. Also, fruit morphology was positively affected, with an increase in the length, diameter, and weight of the fruits in treated plants.

A greenhouse experiment was carried out by Flores-Felix and colleagues, who investigated the effect of the inoculation of the type-strain of *Phyllobacterium endophyticum* (PEPV15) on strawberry plants planted on black plastic trays with peat/vermiculite (Flores-Félix et al., 2015). The results of the experiments showed that the inoculation, six days after planting, with the strain PEPV15 determined an increase in many parameters related to plant growth. Stolons number and length were significantly (p<0.05) higher in the plants inoculated with the strain PEPV15; treated plants also produced a significantly (p<0.05) higher number of flowers and fruits. The latter’s weight was nevertheless not significantly higher (**Table 2**).

Strawberry plants have also been treated in greenhouse experiments in Portugal with three PGPR strains, i.e., *Pedobacter* sp. CC1, *Bacillus safensis* B106 and *B. subtilis* B167A (Morais et al., 2019). Their application allowed for an earlier flowering and harvest time than the control (**Table 2**). A similar positive effect was observed on pear (*Pyrus communis*) seedlings by applying metabolites produced by *P. agglomerans* C1 (Valerio et al., 2023).

However, for economic reasons, it is important that the fruit’s quality is increased, and a plentiful and healthy harvest is guaranteed. Strawberries are soft fruits characterized by a high and rapid loss of firm texture during ripening. The fast-softening results in a shorter shelf-life and higher susceptibility to diseases; it is thus among the main reasons for commercial loss. It is estimated that 5 to 30% of strawberry yield is lost because of over-softening and fungal decay (Posé et al., 2011). Within this framework, it is important to consider the antagonistic properties of PGPR consortia against fungal pathogens.

Regarding strawberries, one of the main postharvest diseases is grey mold, caused by *Botrytis cinerea*. Following its contamination, a grey coating appears on leaves and fruits; the plant dies off, and the fruits become dry and rot. The strawberry plant’s withering is caused by *Verticillium dahliae*, which attacks the plant’s vascular system, blocks the water and nutrients transport, and becomes detrimental to the plant. Drobek and colleagues hence investigated the antagonistic effect of selected bacterial consortia on four microbial pathogens (i.e., *Botrytis cinerea*, *Verticillium* sp., *Phytophthora* sp., and *Colletotrichum* sp.) causing strawberry diseases (Drobek et al., 2021). The bacterial consortia applied comprised strains belonging to *Peanibacillus polymyxa* sp., *B. subtilis*, *Bacillus* sp., *Streptomyces* sp., *Lysobacter* sp., and *Pseudomonas* sp.

The application of PGPR consortia contributed to obtaining fruits with a higher firmness when a PGPR consortium was used as an antagonist agent against a mixed pathogen group composed of *B. cinerea*, *Verticillium* sp., *Phytophthora* sp., and *Colletotrichum* sp. This outcome is of paramount importance because firmness is a property that generally allows for more extended storage periods and makes strawberry fruits more suitable for transport. The increased firmness was likely related to the production of metabolites by the PGPR strains that can limit mycelium growth by bacterial consortia. During ripening, strawberries soften, in fact, following an extensive dissolution of the middle lamella of the cortical parenchyma cells (Posé et al., 2011). The activity of fungal pathogens attacking the strawberry fruit during ripening and storage can increase the degradation. The trend of soluble solid content (SSC) after the treatment was also monitored. This parameter is connected with consumer preference for fruit. SSC measures total soluble solids, including sugars, organic acids, amino acids, and other compounds. The mean SSC in the fruits under analysis was 6%, consistent with the literature (Chen et al., 2018). The treatment of strawberries contaminated with *Phytophthora* sp. with one PGPR consortium also allowed for a higher SSC. The increase in one out of five treatments was likely because some bacteria can dissolve phosphates, which lower soil pH and increase phosphorus availability by producing organic acids. As regards phenolics and anthocyanins, which play a crucial role as bioactive compounds and are also responsible for the bright red color of strawberry fruits, it was observed that total phenolic content was higher than the control only in one treatment. At the same time, total anthocyanin content was 25–51% higher than the control in the strawberry fruits infested by *Phytophthora* sp. and treated with four out of five PGPR consortia.

#### 3.2.2 Effect of microbial PB on strawberry functional and sensory quality

Sugars and organic acids are the main soluble components in ripe strawberry fruit, and the ratio between them affects fruit aroma and flavor (Todeschini et al., 2018). Fructose, glucose, and sucrose are responsible for fruit sweetness (Perez et al., 1997). Organic acids are important for preserving fruit’s nutritional value (Mikulic-Petkovsek et al., 2012). Among them, citric acid is the most abundant and is responsible for about 92% of total acidity. Other organic acids, i.e., malic, tartaric, shikimic, quinic, and fumaric, are present at a very low concentration. Strawberry fruits are also an important source of healthy compounds such as dietary fiber, vitamin C, minerals like potassium and magnesium, and antioxidants. Among polyphenols, anthocyanins are the most important compounds in the form of pelargonidin and cyanidin derivatives. They are, in fact, responsible for the strawberry’s bright red color; in addition, they play a key role in fruit tolerance to environmental stresses and the improvement of post-harvest quality and shelf life. Volatile organic compounds are also important, as they make strawberries a highly appreciated fruit and an important quality indicator for strawberries.

A consortium of three PGPR strains (i.e., *B. subtilis*, *B. amyloliquefaciens*, and *Pseudomonas monteilii*) was evaluated for its potential role as a biofertilizer by application twice a week at three different percentages (0%, 0.24% and 0.48%) to the soil in field experiments (Nam et al., 2023). The three treatments did not significantly affect total acidity (TA), which is a predictor of the impact of organic acids on food flavor and whose content declines during the fruit ripening process (**Table 2**). Regarding total soluble solids (TSS) determination, this parameter was higher in the strawberry sample treated with the highest percentage of biofertilizer (**Table 2**). The strawberry color was also affected by the treatments; it emerged that treated samples were darker than non-treated ones (**Table 2**). Hence, the higher values of TA and TSS and the darker color suggest that the strawberries treated with the biofertilizer were more mature. So, with equal growing periods for all three treatments, data showed that the biofertilizer had a ripening enhancer effect.

The effect of strawberry plant treatment with PGPR strains *B. amyloliquefaciens* BChi1, and *P. fungorum* BRRh-411 was also investigated on antioxidants (Rahman et al., 2018). The application of plant probiotic bacteria significantly increased the total content of anthocyanins, carotenoids, flavonoids, and phenolics, as well as antioxidant activity compared to the non-treated control (**Table 2**).

Vitamin C was detected in the greenhouse experiment carried out by Flores-Felix and colleagues by inoculating the type-strain of *Phyllobacterium endophyticum* (PEPV15) (Flores-Félix et al., 2015). It emerged that its content in strawberry fruits from plants inoculated with strain PEPV15 was significantly higher (i.e., two-fold) than in fruits from non-treated plants (**Table 2**).

In the greenhouse experiment carried out by Todeschini and colleagues with *F. mosseae*, *S. viscosum*, *R. irregularis*, and three strains of *Pseudomonas* sp., it emerged that the treatment with AMF mainly affected the parameters associated with the vegetative portion of the plant. At the same time, the effect of PGPR was significant for fruit yield and quality. Titratable acidity, expressed as the percentage of citric acid per fresh weight unit, was significantly affected by the mycorrhizal treatment and even more by bacterial inoculation (Todeschini et al., 2018). No differences were found in organic acids for quinic, citric, fumaric, and ascorbic acid, whereas malic acid concentration was significantly affected; the treatment with *Pseudomonas* sp. Sv19Fv determined the highest concentration of malic acid. Regarding glucose and total sugars concentrations, the treatments determined non-significant differences, while the effect on sucrose and fructose was related to the combination of AMF and bacterial strains (**Table 2**). As regards anthocyanins, cyanidin 3-glucoside, pelargonidin 3-glucoside, pelargonidin 3-rutinoside, cyanidin malonyl glucoside, pelargonidin malonyl glucoside, and pelargonidin acetyl glucoside were present (**Table 2**). The concentration of pelargonidin 3-glucoside was the most abundant and significantly varied between the treatments (**Table 2**). The various treatments did not significantly affect the concentrations of the other anthocyanidins.

### 3.3 Leafy vegetables

Leafy vegetables are a broad group of horticultural plants cultivated for their foliar structure, constituting the plant’s edible part (Alvino and Barbieri, 2015). The list of most common species of leafy vegetables includes, among others, lettuce (*Lactuca sativa* L.), rocket salad (*Eruca sativa* Miller), and common basil (*Ocimum basilicum* L.) (Alvino and Barbieri, 2015). For these crops, experimental work with PBs has been carried out. The analysis of the main outcomes of these studies is of paramount importance because consumption of salads and herbs such as basil is particularly high in the Mediterranean countries where the cultivation of the species has ancient traditions.

#### 3.3.1 Lettuce

Lettuce (*Lactuca sativa* L.) is a leafy vegetable belonging to the *Cicoreae* tribe of the family *Compositae*. It is an excellent source of vitamin A and K, provitamin A compounds, and beta-carotene, in darker green lettuces, such as Romaine (Kim et al., 2016). It is also a good source of folate and iron and has an interesting phytochemical profile. Lettuce comes in various colors, sizes, and shapes, and because of this diversity, it can be grouped into diverse types. According to Mou (2008), six main lettuce types are identified based on leaf shape, size, texture, head formation, and stem type: i) crisphead lettuce (var. *capitata* L. *nidus jaggeri* Helm), ii) butterhead lettuce (var. *capitata* L. *nidus tenerrima* Helm), iii) romaine or cos lettuce (var. *longifoli*a Lam., var. romana Hort. in Bailey), iv) leaf or cutting lettuce (var. *acephala* Alef., syn. var. *secalina* Alef., syn. var. *crispa* L.), v) stem or stalk (Asparagus) lettuce (var. *angustana* Irish ex Bremer, syn. var. *asparagina* Bailey, syn. L. *angustana* Hort. In Vilm.), and vi) Latin lettuce (Mou, 2005, Mou, 2008).

Iceberg lettuce was treated with *Trichoderma*-based biostimulants under sub-optimal, optimal, and supra-optimal nitrogen (N) fertilization levels and grown in a greenhouse (Fiorentino et al., 2018). *Trichoderma* strains *T. virens* (GV41), or *T. harzianum* (T22) were used in the inoculation. Thanks to the treatment, no visible chlorosis or necrosis symptoms were observed in treated plants. The yield was positively affected by the treatment with both strains and the supply of an optimal dose of nitrogen; moreover, no significant effect was observed between the two strains. When the soil was not fertilized with N, the inoculation of the GV41 strain gave better results than T22 for the total and marketable weight (**Table 3**). Ascorbic acid is an important parameter of the functional quality of horticultural crops, and the study by Fiorentino et al. (2018) showed that the *Trichoderma*-based biostimulants significantly influenced it, N availability rate and interaction thereof: the highest concentration was observed whatever strain was inoculated under no-fertilization conditions.

**Table 3:** Conditions and effect of microbial plant biostimulants treatment on leafy vegetables.

Crop	Experiment Type and Location	PGPR	AMF	Quality parameter	Treatment Effect	Reference
Lettuce (*Lactuca sativa* L. var. Iceberg cv. Silvinas)	Greenhouse experiment (Italy)	–	*Trichoderma virens* GV41 *T. harzianum* T22	Total yield	↑ (optimal N) ↑ (GV41+no N supply)	(Fiorentino et al., 2018)
				Marketable yield	↑ (optimal N) ↑ (GV41+no N supply)	
				pH	=	
				Ascorbic acid	↑ (non-fertilized conditions)	
Leaf lettuce (*Lactuca sativa* var. *crispa* L. cv. Santoro)	Field experiment (Czech Republic)	*Bacillus licheniformis* *Bacillus megatherium* *Azotobacter* sp. *Azospirillum* sp. *Herbaspirillum* sp.	–	Leaf weight	↑	(Kopta et al., 2018)
				TAA	=	
				TCC	=	
Romaine lettuce (*Lactuca sativa* L. var. *longifolia* Lam. cv. Quintus)	Field experiment (Czech Republic)	*Bacillus licheniformis* *Bacillus megatherium* *Azotobacter* sp. *Azospirillum* sp. *Herbaspirillum* sp.	–	Leaf weight	↑	(Kopta et al., 2018)
				TAA	↑	
				TCC	=	
Butterhead lettuce (*Lactuca sativa* var. *capitata* cv. Bolla)	Greenhouse experiment (Italy)	–	*R. irregulare* *F. mosseae* *T. koningii*	AMF/*Trichoderma* presence	↓	(Saia et al., 2019)
				Plant yield	↑	
				P, Mg, Fe, Mn, Zn, Ca, Cu	↑	
				Phenolic compounds	↑	
				Luteolin glycoside	↑	
Rocket (*Eruca sativa* Mill.)	Greenhouse experiment (Italy)	–	*Trichoderma virens* (strain GV41) *T. harzianum* (strain T22)	Marketable yield	= (optimal N)	(Fiorentino et al., 2018)
				Total yield	↑ (GV41)	
				pH	=	
				Ascorbic acid	↑ (GV41)	
Basil (*Ocimum basilicum* L. Gecom)	Glasshouse experiment (Italy)	–	AMF and *T. koningii*	Leaf number	↑	(Saia et al., 2021)
				Leaf area	↑	
				Plant photosynthetic activity	↑	
				Ca, Mg, B concentration	↑	
				p-Coumaric acid	↑	
				Chicoric acid	↑	

↑: increase; ↓: decrease; ↑↓: variable; =: no significant differences.

In the same experiment, the *Trichoderma* biostimulant was also applied to the rocket (*Eruca vesicaria* Mill.), but the inoculation had a more beneficial effect on iceberg lettuce than the rocket. Among the different reasons, including also the fact that crop response is not global but complex and generally depends on the crop botanical family, it is likely that the lettuce crop cycle was longer. This means that the higher duration allowed the root system to develop and diversify more than the rocket, following a major ability of the AMF to colonize the crop rhizosphere.

Saia and colleagues investigated the response triggered by a microbial PB containing two strains of AMF (i.e., *R. irregulare* and *F. mosseae*) and *Trichoderma koningii* on greenhouse-grown lettuce (*Lactuca sativa* L.). The experiment was performed under different water conditions: i) well-watered, ii) moderate-watered, and iii) severe deficit irrigation regimes. The study outcomes suggested that the effect of the biostimulant on lettuce’s nutritional and functional quality was mainly independent of water availability. In contrast, its effect on fresh marketable and dry yields was clear under well-watered and moderate irrigation regimes through the modulation of the secondary compounds’ biosynthesis (Saia et al., 2019). Under the well-watered and moderate irrigation regimes, yield, phenolic acids, and flavonoids were not affected, whereas net photosynthetic and transpiration rates were halved. In addition, the presence of AMF and Trichoderma reduced Mg and Zn concentrations in the roots, soil, and plant. After further reducing water availability, yields, ascorbic acid, total phenols, and quercetin also decreased. However, the treatment increased P, Mg, Fe, Mn, and Zn concentrations and various phenolic acids, such as chlorogenic acid, irrespective of the water availability. The increase in plant yield, calcium, copper, and isochlorogenic acid concentration was especially evident under well-watered and moderate irrigation conditions. Finally, luteolin glycoside, which is directly implied in the neighbor detection and allelopathy response of plants and is frequently associated with a plant reaction to drought stress and microbial stimulation, progressively increased in the biostimulant inoculated plant upon water availability reduction.

Leaf lettuce (*Lactuca sativa* var. *crispa* L.) cv. ‘Santoro’ and Romaine lettuce (*Lactuca sativa* L. var. *longifolia* Lam.) cv. ‘Quintus’ was treated (watering) with some PGPR strains, i.e., *Bacillus licheniformis*, *Bacillus megatherium*, *Azotobacter* sp., *Azospirillum* sp., *Herbaspirillum* sp., and freshwater algae (*Chlorella vulgaris*), in a field experiment. The two lettuce varieties were cultivated at different times: spring and summer. Considering a mean trend, the weight of leaf and romaine lettuce-treated plants was significantly higher than the control (**Table 3**). As regards the effect of the treatment on total antioxidant capacity and total carotenoid content, no significant effect was observed between treated plants and control. However, total antioxidant capacity for the spring and summer crops was generally higher in leaf lettuce than in romaine.

#### 3.3.2 Rocket

Rocket (*Eruca sativa* (Mill.) Thell) is a leafy green vegetable from the *Brassicaceae* family. It contains many vitamins, including vitamins A and C and folic acid (Jamal et al., 2021). It is also rich in several minerals, such as calcium, copper, iron, magnesium, phosphorus, potassium, and zinc. Rocket is well-known for its various phytochemical components, including polyphenols, flavonoids, and glucosinolates (Jamal et al., 2021).

In the same greenhouse experiment described for iceberg lettuce, Fiorentino et al. (2018) investigated the effect of inoculation with *T. virens* strain GV41 and *T. harzianum* strain T22 also on the rocket (*Eruca vesicaria* Mill.). Under sub-optimal N fertilization conditions, treatment with *T. virens* GV41 determined a 33% increase in the total yield rocket (**Table 3**). In rocket, ascorbic acid presented the same trend as in iceberg lettuce. Its concentration was significantly affected by the *Trichoderma*-based biostimulants, N availability rate, and interaction thereof (Fiorentino et al., 2018). In detail, inoculation with strain GV41 under N fertilization treatments determined a significant increase in total ascorbic acid compared to T22 (**Table 3**).

The increase in rocket plant growth and productivity under the three fertilization conditions hints that *Trichoderma* can modify soil nutrient availability, modulate root growth, and subsequently affect the rhizosphere’s various biological and chemical processes. Some strains of the *Trichoderma* species produce, in fact, secondary metabolites, which have a hormone-like behavior; hence, the exudation of molecules with auxin-similar activity has a plant growth promotion action (Fiorentino et al., 2018). *T. harzianum* strain T22 is not a remarkable producer of bioactive compounds which can stimulate plant growth. However, its application to the rhizosphere can activate plant metabolic processes involving phytohormones (auxins/cytokinins) in treated plants.

All in all, the effect of the treatment was less beneficial on rockets than on lettuce plants. As mentioned above, the rocket is a leafy green vegetable belonging to the Brassicaceae family, and Brassicaceae species are well-known for the adverse effects on many soil microbes, bacteria, and fungi following the production of inhibitory compounds such as glucosinolates, which are released in the rhizosphere (Fiorentino et al., 2018).

#### 3.3.3 Basil

Basil is widely cultivated in pots and gardens in Europe, South-west Asia, and the USA. It is one of the most popular herbs in the cooking and food network with its wide range of applications, especially in food flavoring and preservation. The leaves are ovate and vary in size, depending on the variety; they range from the small leaves of common basil to the large leaves of lettuce leaf basil (Kokkini et al., 2003). Saia et al. (2021) demonstrated that genotype, cultivation medium, and growing conditions affect several basil properties, including i) tolerance to salinity, which is generally low; ii) parameters of economic importance, such as the leaf fraction on the total above-ground biomass; iii) the concentration, and composition of secondary compounds; the antioxidant capacity; iv) the volatile organic fractions; v) the essential oil content. The same authors evaluated, in greenhouse experiments, the effectiveness of a microbial-based biostimulant containing two strains of AMF (i.e., *R. irregulare* BEG72 and *F. mosseae* BEG234) and *T. koningii* on the growth of basil under mild salinity conditions: 25 mM (low salinity) and 50 mM (high salinity). The increase in the salinity showed detrimental effects on the plant yield, nutrient uptake and concentration, photosynthetic activity, and leaf water potential, whereas it triggered the polyphenols accumulation. The concentration of eucalyptol and β-linalool, two of its main essential oil constituents, also decreased (Saia et al., 2020). However, the inoculum showed a beneficial effect on plant growth, leaf number, and area, irrespective of the salinity stress condition; Ca, Mg, B, *p*-coumaric and chicoric acids also accumulated. The results suggested that under low-salinity conditions, the inoculum stimulated the plant’s photosynthetic activity following a higher availability of iron and manganese for the plant and subsequently induced the accumulation of phenolic acids, such as caffeic and rosmarinic acids. Under high salinity conditions, the inoculum mostly sequestered Na and increased P availability for the plant; moreover, it stimulated the accumulation of some polyphenols (e.g., ferulic and chicoric acids and quercetin-rutinoside) in the shoots. The inoculum did not affect the composition of volatile organic compounds, thus suggesting the lack of interaction between its activity and essential oil biosynthesis.

### 3.4 Cucumbers

Cucumber (*Cucumis sativus* L.) is a member of the *Cucurbitaceae*, and among the 30 species of Cucumis, *C. sativus* has the greatest economic significance (Zieliski et al., 2016). It is very rich in water (about 96.4%) and contains other bioactive compounds, such as dietary fiber, vitamin C, phenolic compounds (e.g., flavonols and proanthocyanidins) (Zieliski et al., 2016). During cucumber fruit growth, malic acid content decreases; glucose and fructose content increases; dry matter decreases. Cucumber presents a variation in the color of ripened fruit, fruit size, and number of fruit spines (Schaffer and Paris, 2016). Fruits mainly differ in the length and not width; hence, fruit size is essentially a function of length (Schaffer and Paris, 2016).

Cucumber plants can tolerate 3% salinity stress (Ge and Zhang, 2019), and, under salinity stress conditions, photosynthetic pigments, chlorophyll synthesis, plant metabolic and physiological activities are negatively affected because primary and secondary metabolites fluxes are altered (Abdel-Farid et al., 2020). Kartik et al. thus investigated the possibility of applying microbial PBs to cucumber crops as a possible approach to tackle the abiotic stress of salinity stress (Kartik et al., 2021). Treatments were made under a saline stress condition and with no saline stress. The study showed that plant growth parameters, such as root length, root fresh and dry weight, shoot length, and shoot fresh and dry weight, generally increased compared to control plants under both growth conditions. Differences emerged depending on the applied PGPR strain (**Table 4**). The measured plant growth parameters, except for root length and dry weight, were significantly higher under salinity stress conditions than the control (**Table 4**). The enhancement of plant morphological traits by treating the salt tolerant PGPR may be related to their ability to produce phytohormones and solubilize available minerals in the soil. The production of indole-3-acetic acid and siderophores and the ability of N and P uptake by the applied PGPR may have also played a crucial role in fostering plant growth under salinity stress.

**Table 4:** Conditions and effect of microbial plant biostimulants treatment on cucumbers.

Crop	Experiment Type and Location	PGPR	AMF	Quality parameter	Treatment Effect	Reference
Cucumber (*Cucumis sativus* L.)	Pot experiments (India)	*Acinetobacter baumannii* (2 strains) *Arthrobacter* sp. *Cronobacter dublinensis* (4 strains) *Enterobacter cloacae*	–	Root length	↑ (5 out of 8, no saline stress) ↑ (2 out of 8, saline stress)	(Kartik et al., 2021)
				Root wet weight	↑ (3 out of 8, no saline stress) ↑ (4 out of 8, saline stress)	
				Root dry weight	↑ (7 out of 8, no saline stress) ↑ (2 out of 8, saline stress)	
				Shoot length	↑ (5 out of 8, no saline stress) ↑ (7 out of 8, saline stress)	
				Shoot wet weight	↑ (3 out of 8, no saline stress) ↑ (7 out of 8, saline stress)	
				Shoot dry weight	↑ (4 out of 8, no saline stress) ↑ (7 out of 8, saline stress)	
				Chlorophyll a content	↑ (4 out of 8, no saline stress) ↑ (6 out of 8, saline stress)	
				Chlorophyll b content	↑ (4 out of 8, no saline stress) ↑ (8 out of 8, saline stress)	
				Ascorbic acid	↑ (4 out of 8, no saline stress) ↑ (8 out of 8, saline stress)	
				TPC	↑ (8 out of 8, no saline stress) ↑ (8 out of 8, saline stress)	

TPC, Total Phenolic Content; ↑: increase.

On the other hand, the beneficial effect on chlorophyll is related to induced systemic tolerance by PGPR under salinity stress. The applied PGPR isolates also showed the production of siderophores, HCN, and chitinase. Thus, the strains likely have biocontrol ability and can protect plants against pathogens.

## 4 Evaluation of the effect of microbial PBs on horticultural crops by omic sciences

The biostimulatory effect exerted by the different microbial PBs presented above derives from the interaction between the molecular structure of plant cells and a series of external physical, chemical and biological stimuli (González-Morales et al., 2021). Plants can, in fact, adapt to environmental conditions by exerting physiological and biochemical responses and by modifying metabolic processes. It is therefore within this framework that “omic” sciences (e.g., genomics, transcriptomics, proteomics, and metabolomics) have figured out as fundamental tools for decoding and unravelling the metabolic pathways and the key factors underlying the plant response to both endogenous and environmental stimuli.

The application of “omic” sciences to disentangle the mode(s) of action by which microbial PBs affect plant quality has turned out as informative and useful; however, a limited number of studies is still available.

Transcriptomic analysis, based on Next-Generation Sequencing, figured out as one of the most powerful tools allowing for the identification of the molecular markers that are associated with common responses of plants to the application of microbial PBs. Transcriptomic analysis has been performed to understand the mode of action of different PBs, such as fungi (Volpe et al., 2018), biostimulants based on humic acids and chitosan (Hernández-Hernández et al., 2018), biostimulants based on extracts of algae and botanicals (Goñi et al., 2018), and commercial products (Contartese et al., 2016) on horticultural crops such as tomato, lettuce and cucumbers. As regards tomato cropping, a transcriptomic approach is proposed only in a few studies on microbial PBs. The application of *Trichoderma harzianum* T22 to tomato seedlings showed that, under water stress, the strain modulates the expression of genes encoding some antioxidant enzymes, such as monodehydroascorbate reductase (MDHAR), glutathione reductase (GR), dehydroascorbate reductase (DHAR) (Mastouri et al., 2012). The genes MDHAR1 and MDHAR2, GRc and GRp, DHARc and DHARp, and Cu/Zn-SOD*p*, Fe-SOD*p*, and APX*c* showed a higher expression in the tomato shoots, while the gene APX*c* showed a higher expression in tomato roots (Mastouri et al., 2012). These findings hint at the fact that an enhanced resistance of treated plants to water stress is due to the increased capacity to scavenge reactive oxygen species and recycle oxidized ascorbate and glutathione. The expression of genes under water deficit was also studied following the application of *Funneliformis mosseae* and *Rhizophagus intraradices* to a commercial tomato cultivar (San Marzano nano) (Volpe et al., 2018). An improvement of phosphorous uptake, transfer and delivery emerged in tomato roots, and the genes encoding for plant phosphate transporters (i.e., LePT1, LePT2, LePT3, LePT4, and LePT4) changed their expression following the treatment with AMF and under water deficit.

Genes related to plant growth promotion and biocontrol activity in tomato seedlings were found in the genome of *Pantoea agglomerans* strain C1 (Luziatelli et al., 2020b). In detail, study identified i) two genes, namely, *ipdC* and *amiE*, which encode key enzymes (i.e., indole-3-pyruvate decarboxylase and aliphatic amidase) involved in the synthesis and secretion of inole-3-acetic acid via the IPyA and IAM pathway, respectively; ii) two operons (i.e., speAB and speDE) involved in the biosynthesis of spermidine and correlated with the development of lateral roots, resistance to pathogens, as well as resistance to oxidative, osmotic, and acidic stress; and iii) several gene clusters involved in the solubilization of mineral phosphate.

As regards to the other horticultural crops analyzed in this study, it emerged that “omic” sciences have been so far applied to understand the response of plants to PBs other than microbial ones. A transcriptomic approach has been used, for instance, to understand the response of lettuce seedlings to microalgae extracts used as biostimulant agents (Santoro et al., 2023). Otherwise, the proteomic changes occurring in the antioxidant system of strawberry during ripening was investigated (Song et al., 2020). The investigation of a total of 46 proteins and isoforms by a targeted quantitative proteomic approach using multiple reaction monitoring, showed that superoxide dismutase, aldo/keto reductase, and glutathione transferase increased significantly, whereas L-ascorbate peroxidase, 1-Cys peroxiredoxin, 2-Cys peroxiredoxin, dehydroascorbate reductase, and catalase decreased significantly. These plants were not treated with biostimulants. However, a meaningful interpretation of such analyses requires reproducible effects. It might be, therefore, interesting investigating these markers in plant treated with microbial PBs.

Besides the increasing need to use “omic” sciences to understand the mechanisms that affect food quality following plant treatment with microbial biostimulants, high-throughput phenotyping technologies are necessary to identify the optimal phenological stage, the application method, time, and rate that allow improving plant performance and resilience to stress (Rouphael and Colla, 2018). They offer the advantage of an automated and non-destructive monitoring of the morpho-physiological traits of plants, as well as the possibility to carry out time-series measurements which provide information on growth progression, plant performance and stress response (Rouphael and Colla, 2018). In addition, these technologies can reduce costs, labor and analysis time (Rouphael and Colla, 2018), but are also prone to produce artefacts, when a meaningful interpretation with common human sense is missing.

## 5 Concluding remarks and future perspectives

This study identified the various effects that microbial PBs could have on plant and fruit morphology, crop productivity, and fruits’ nutritional, functional, and organoleptic quality. Currently, most studies occurred under greenhouse conditions, where it is likely that growing conditions can be better controlled and monitored. Field trials were carried out only in a small amount. Tomato, lettuce, and basil crops have been primarily treated with AMF, while PGPR metabolites were used for other crops, such as strawberries and cucumbers.

Findings showed that crop response is never univocal and global. Complex mechanisms related to the PB type, the strain, and the crop botanical family, occur. It is necessary to continue designing other experiments where the mechanisms behind the plant growth-promoting activity are also tracked. These observations indicate the critical points to be considered in developing new PBs. First-generation PBs were produced with bioactive substances and microorganisms to stimulate plant physiological and molecular processes and improve plant nutrient uptake and use efficiency. More recently, a second generation of PBs has been formulated thanks to the new synergistic work of chemistry, biology, and omics sciences, but the importance of agricultural and horticultural management issues is still underestimated. For the rational development of the third generation PBs, we need to better understand the molecular mechanisms allowing the modulation of plant physiology and the synergistic effect of microbial and non-microbial biostimulants. A more comprehensive knowledge of the mechanisms underlying the biostimulant activity will allow identifying the best-suited biostimulant for a specific crop and exact growing conditions. In addition, a more profound knowledge of the molecular mechanisms will be valuable for identifying the optimal dose to apply and the suitable stage of plant development and growth at which a specific biostimulant should be applied. Finally, more extensive and comprehensive knowledge, which also requires an adequate legal framework regarding efficacy testing, risk assessment and registration procedures with the respect to the actual nature of the regulated agents (Feldmann et al., 2022), might allow biostimulant manufacturers to address the product composition with a major awareness.

## Author contributions

FM and VM contributed to the conceptualization and definition of the methodology and investigation of the study. MR contributed to defining the design of the study. FM and VM wrote the first draft of the manuscript. FM, VM, FL, RJ, AF, MR contributed to editing the final version of the manuscript. All authors contributed to the article and approved the submitted version.
